# Diagnosis and management of an immature teratoma during ovarian stimulation: a case report

**DOI:** 10.1186/1752-1947-5-540

**Published:** 2011-11-04

**Authors:** Nathalie Douay-Hauser, Martin Koskas, Francine Walker, Dominique Luton, Chadi Yazbeck

**Affiliations:** 1 Obstetrics, Gynecology and Reproductive Medicine Department, Bichat Claude Bernard University Hospital, 46, rue Henri-Huchard, 75018 Paris, France; 2 Pathological Anatomy and Cytology Department, Bichat Claude Bernard University Hospital, 46, rue Henri-Huchard, 75018 Paris, France; 3 INSERM UMRS 1018 - CESP, 16, avenue Paul-Vaillant-Couturier, 94807 Villejuif, France

## Abstract

**Introduction:**

The discovery of a mature teratoma (dermoid cyst) of the ovary during ovarian stimulation is not a rare event. Conversely, we could not find any reported cases of immature teratoma in such a situation. Clinical and ultrasound arguments for this immature form are scarcely or poorly evaluated.

**Case Presentation:**

We describe the case of a 31-year-old Caucasian woman with primary infertility, who developed an immature teratoma during an in vitro fertilization ovarian stimulation cycle.

**Conclusions:**

Ultrasound signs of an atypical cyst during ovarian stimulation allowed us to adopt a careful medical attitude and to adapt the required surgical oncological treatment.

## Introduction

Ovarian teratomas represent 15% to 20% of ovarian germ cell tumors. The immature form was first described in 1960 by Thürlbeck and Scully, and can be pure or mixed with a mature component [1]. It is encountered in about 1% of all ovarian teratomas. To the best of our knowledge, no cases of immature teratomas have been described during ovarian stimulation for In Vitro Fertilization (IVF) cycles.

## Case presentation

A 31-year-old Caucasian woman with no particular history, consulted for primary infertility. Basal hormonal tests showed a decrease in ovarian reserve. Cycle day three ultrasound examination counted four antral follicles in both ovaries, without any suspicious cystic lesion. Hysterosalpingography and male sperm test results were satisfactory.

Ovarian stimulation for IVF was started according to the antagonist protocol with human menopausal gonadotropins (hMG) 300 IU/day. Pelvic ultrasound on day 11 revealed a 23 mm anechoic cyst on the left ovary. On day 13, the observed cyst had increased in size (45 mm), was highly vascularized and had a heterogeneous appearance. Nevertheless, it was decided to proceed with ovulation induction. During oocyte retrieval on day 15, the left ovarian cyst measured 82 × 63 × 62 mm, with mixed echogenecity. Color Doppler showed richly vascularized intracystic tissue vegetations. No associated peritoneal effusion was observed.

It was decided not to puncture the left ovary. Four oocytes were retrieved from the right side and all four embryos obtained were frozen at the pronuclear stage.

Our patient was scheduled for prompt surgical treatment, but before that occurred she presented with left abdominal tenderness with suspected adnexal torsion to the emergency ward. This condition necessitated emergency laparoscopy. A 12 cm ovarian cyst with uniform wall was excised. There were no extra cystic vegetations or peritoneal effusion or granulations. Serum tumor markers CA19.9 and CA125 were elevated at 56 U/mL (normal < 40) and 215 U/mL (normal < 35), respectively.

**Figure 1:**
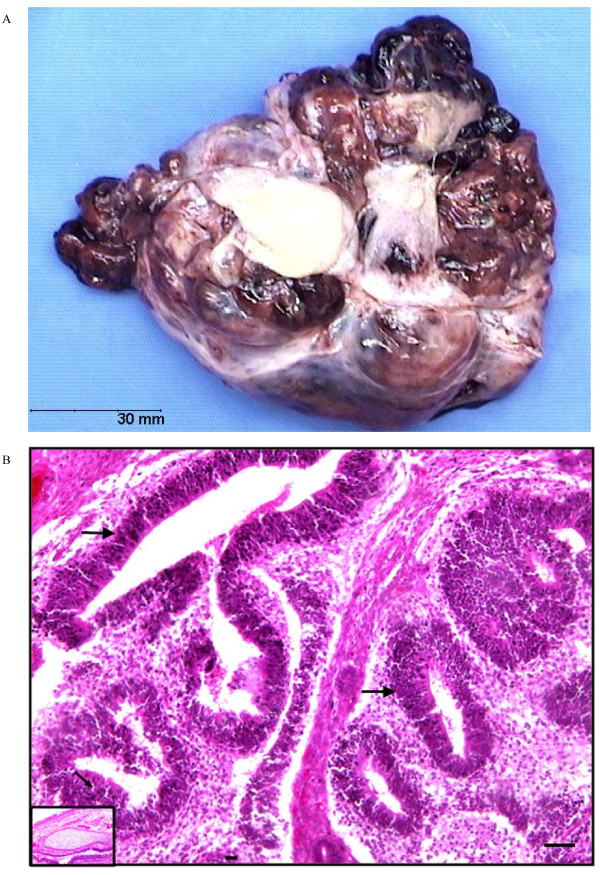
**Immature Teratoma during ovarian stimulation**. **A**: Macroscopic aspect: large irregular cyst with prominent solid component. The cystic areas are filled with fatty sebaceous material. No extra cystic vegetations. **B**: Pathological findings: Immature teratoma with nerve tissue of the embryonic type composed of glial tissue and neuro-ectodermal rosettes of 'primitive neural-tube' type (arrows). **Inset**: Pluritissued mature teratoma sector with cartilaginous tissue.

Pathological examination revealed an immature ovarian teratoma, with a grade 2 neuroectodermal contingent according to Thurlbeck and Scully's histoprognostic scoring as modified by Norris *et al*. [2] (Figure 1). There were several areas composed of abundant immature nervous and glial tissues. Immunohistochemistry revealed S100 protein, synaptophysin and anti-Glial Fibrillary Acidic protein antibodies which marked immature nervous and glial tissues. Peritoneal cytology was negative. The patient was at FIGO stage IA.

The multidisciplinary cancer team authorized a fertility-sparing management. We conducted a second-look laparoscopy for staging, oophorectomy and multiple biopsies, and discovered peritoneal granulations corresponding to peritoneal gliomatosis which was confirmed by the presence of mature glial tissue revealed on histology. Since initial staging was not modified, she had no adjuvant chemotherapy but received regular surveillance by tumor markers, ultrasonography and abdominopelvic computed tomography. Her clinical condition was stable. As a precaution, ovarian stimulation was discouraged.

Four thawed embryos were transferred 10 and 12 months later on two spontaneous cycles but no pregnancy was obtained. Three years after the initial diagnosis, she had no clinical symptoms.

## Discussion

This is a case of a rapidly developing immature teratoma during ovarian stimulation. Immature teratomas are usually derived from a malignant transformation of mature teratomas [3,4]. The amount of neuroectodermal immature tissue present permits the classification of immature teratomas into three grades of increasing malignancy. Peritoneal gliomatosis consists of mature glial tissue implants in the peritoneum and is rarely present [5].

We did not find any reported cases of immature teratomas that occurred during ovarian stimulation. Such teratomas are usually diagnosed in younger patients who have a low probability of using fertility treatments. Although IVF does not seem to have any effect on mature cystic teratomas [6], the possible role of hormonal therapy remains highly suspected in this case: the histological findings in our patient did not reveal any component usually sensitive to follicle-stimulating hormone and luteinizing hormone. No estradiol or progesterone specific receptors were expressed on immunohistochemistry. Nevertheless, the rapid development of the cyst that was not identified just before ovarian stimulation suggests otherwise.

The richly vascularized color Doppler aspect is an important element which, combined with the rapid growth of this tumor, was one of the major signs suggesting the malignancy of this cyst.

Conservative treatment of immature teratoma is possible, and does not seem to influence recurrence and survival rates. Furthermore, this tumor is highly chemo-sensitive. Successful medically assisted pregnancies have been reported after fertility sparing surgical management followed by cisplatin, etoposide and peplomycin chemotherapy [7]. Sterility may still be observed in advanced stages associated with rapidly growing tumors where oophorectomy is mandatory. In these cases, it is advisable to consider cryopreservation of oocytes or embryos before treatment [4].

## Conclusions

This brief report highlights the potential role of ovarian stimulation on the development of ovarian germ cell tumors, which requires fertility specialists to apply absolute rigor in the management of any cystic mass appearing before or during hormonal treatment. Thorough ultrasound screening is mandatory in every woman under ovarian stimulation. Such an attitude could help to avoid operating on ovarian tumors with delay or without necessary precautions.

## Consent

Written informed consent was obtained from the patient for publication of this case report and accompanying images. A copy of the written consent is available for review by the Editor-in-Chief of this journal.

## Competing interests

The authors declare that they have no competing interests.

## Authors' contributions

NDH analyzed and interpreted the patient data and was a major contributor in writing the manuscript. CY performed the medical treatment. CY and MK performed surgical treatment. FW performed the histological examination of the ovary. DL and all authors read and approved the final manuscript.
